# Gene Expression of Sirtuin-1 and Endogenous Secretory Receptor for Advanced Glycation End Products in Healthy and Slightly Overweight Subjects after Caloric Restriction and Resveratrol Administration

**DOI:** 10.3390/nu10070937

**Published:** 2018-07-21

**Authors:** Alessandra Roggerio, Célia M. Cassaro Strunz, Ana Paula Pacanaro, Dalila Pinheiro Leal, Julio Y. Takada, Solange D. Avakian, Antonio de Padua Mansur

**Affiliations:** Instituto do Coração, Hospital das Clínicas—HCFMUSP, Faculdade de Medicina, Universidade de São Paulo, Av Dr Eneas de Carvalho Aguiar, 44. CEP 05403-900 São Paulo, SP, Brazil; alessandra.roggerio@incor.usp.br (A.R.); labcelia@incor.usp.br (C.M.C.S.); ana.pacanaro@incor.usp.br (A.P.P.); dalila.pinheiro.leal@hotmail.com (D.P.L.); jyt@bol.com.br (J.Y.T.); solange.avakian@incor.usp.br (S.D.A.)

**Keywords:** resveratrol, caloric restriction, esRAGE, Sirt-1

## Abstract

Sirtuin-1 (Sirt-1) and an endogenous secretory receptor for an advanced glycation end product (esRAGE) are associated with vascular protection. The purpose of this study was to examine the effects of resveratrol (RSV) and caloric restriction (CR) on gene expression of Sirt-1 and esRAGE on serum levels of Sirt1 and esRAGE in healthy and slightly overweight subjects. The study included 48 healthy subjects randomized to 30 days of RSV (500 mg/day) or CR (1000 cal/day). Waist circumference (*p* = 0.011), TC (*p* = 0.007), HDL (*p* = 0.031), non-HDL (*p* = 0.025), ApoA1 (*p* = 0.011), and ApoB (*p* = 0.037) decreased in the CR group. However, TC (*p* = 0.030), non-HDL (*p* = 0.010), ApoB (*p* = 0.034), and HOMA-IR (*p* = 0.038) increased in the RSV group. RSV and CR increased serum levels of Sirt-1, respectively, from 1.06 ± 0.71 ng/mL to 5.75 ± 2.98 ng/mL (*p* < 0.0001) and from 1.65 ± 1.81 ng/mL to 5.80 ± 2.23 ng/mL (*p* < 0.0001). esRAGE serum levels were similar in RSV (*p* = NS) and CR (*p* = NS) groups. Significant positive correlation was observed between gene expression changes of Sirt-1 and esRAGE in RSV (*r* = 0.86; *p* < 0.0001) and in CR (*r* = 0.71; *p* < 0.0001) groups, but not for the changes in serum concentrations. CR promoted increases in the gene expression of esRAGE (post/pre). Future long-term studies are needed to evaluate the impact of these outcomes on vascular health.

## 1. Introduction

Sirtuin-1 (Sirt-1) and an endogenous secretory receptor for an advanced glycation end product (esRAGE) are associated with vascular protection. Sirt1 plays an important role in vascular biology and regulates aspects of age-dependent atherosclerosis. In mammals, there are seven sirtuin isoforms from Sirt-1 to Sirt7. Sirt1 is found predominantly in the cell nucleus and has a number of modulators such as polyphenolic activators (resveratrol). Animal models confer cardio-protection, reduce neurodegeneration, promote increased fatty acid oxidation and gluconeogenesis in the liver, reduce lipogenesis in the white adipose tissue, and increase insulin secretion in the pancreas and insulin sensitivity in the muscle [1]. Sirt1 through stimulation of nitric oxide synthase promotes vascular vasodilation, endothelium regeneration, and cardiomyocyte protection under stressful conditions and cellular toxicity to reactive oxygen species [2,3]. Caloric restriction (CR) and resveratrol (RSV) are two interventions associated with higher gene expression and serum concentrations of Sirt-1 in animal studies [4,5] and in humans [6,7]. Studies have shown that increased concentrations of Sirt-1 are associated with better vascular homeostasis and metabolic profile and protection against endothelial senescence [8,9]. The receptor for advanced glycation end-products (RAGE) is a multi-ligand receptor for the final products of non-enzymatic glycation termed advanced glycation end products (AGEs) and expressed in alveolar epithelial cells of the lung and in endothelial and smooth muscle vascular cells [10]. Overconsumption of dietary AGEs causes chronic high-oxidative stress and inflammation and induces diabetic vasculopathy [11]. Bacon, processed beef, chicken, oils (olive and peanut), and cheeses (parmesan, American, and feta) are primary dietary source of AGEs [12]. Overexpression of RAGE has been associated with atherosclerosis and diabetic vascular diseases [13]. In prediabetic patients, AGEs were associated with the down-regulation of Sirt-1 expression and enzyme activity [14]. RAGE undergoes extensive alternative splicing to produce a variety of transcripts from a single gene. Alternative splicing produces different RAGE protein isoforms with diverse functions. Two major splicing variants have been characterized. Membrane bound RAGE is also known as a full-length RAGE (flRAGE) and esRAGE is a circulating truncated variant of the RAGE isoform [15]. esRAGE acts as a soluble antagonist that competes with cell surface RAGE as a receptor scavenger for circulating AGEs and reducing their availability for RAGE receptors located in the cell membrane. This decreases the harmful effects on cells. Studies have shown that low plasma concentrations of esRAGE is associated with the risk of diabetes, coronary artery disease, and all-cause mortality [16,17]. The purpose of this study was to examine the effect of RSV consumption, CR on Sirt-1, RAGE expression, and serum concentration in healthy and slightly overweight subjects.

## 2. Materials and Methods

The trial design has been described elsewhere [7]. The trial was a prospective randomized trial conducted in 48 healthy subjects from 55 to 65 years of age. The subjects were sedentary or on light physical activity. The subjects were recruited consecutively based on their normal clinical history, physical examination, and normal resting electrocardiogram. After a period of washout of 15 days without the use of any medications or supplements, 24 men and 24 women after menopause (01 year of natural amenorrhea) were randomized to CR or RSV groups. Twenty-four subjects (12 women and 12 men) were prescribed a low-calorie diet (1000 calories/day) and the remaining 24 subjects (12 women and 12 men) received 500 mg of resveratrol (trial registration: http://www.ClinicalTrials.gov; identifier:NCT01668836). Exclusion criteria were BMI ≥ 30 kg/m^2^, smokers, hypertension (using antihypertensive medication or diastolic blood pressure ≥ 90 mmHg), dyslipidemia (use of lipid-lowering medication or serum triglyceride levels ≥ 150 mg/dL or total cholesterol ≥ 240 mg/dL), fasting glucose ≥ 110 mg/dL or using hypoglycemic medication, hormone replacement therapy, premenopausal women, and any other self-reported history or treatment for chronic renal failure (serum creatinine ≥ 2.0 mg/dL), liver failure, or metabolic clinically significant endocrine, hematologic, and respiratory factors. Clinical characteristics and laboratory tests were obtained before the interventions and 30 days after the interventions. The main clinical features analyzed were age, sex, BMI, waist circumference, blood pressure, and heart rate. All participants provided written informed consent for study participation. The Ethics Committee of the University of São Paulo Medical School approved the study (CAAE:00788012.8.0000.0068).

### 2.1. Interventions

The CR dietary intervention was a standard diet of 1000 calories from our Department of Nutrition, which corresponded to a reduction of around 50% of the daily caloric intake of the study subjects. A food nutritional control diary was also used to analyze adherence to the proposed diet. Subjects were instructed to write down all ingested food on a day-by-day basis. A daily food record was not used in the RSV group. RSV was administered 500 mg/day (250 mg twice a day) to the RSV study group. The capsules were obtained from a manipulation pharmacy (Buenos Ayres Pharmacy, São Paulo, Brazil). The purity of the product supplied was analyzed by capillary electrophoresis using the Proteome Lab PA800 from Beckman Coulter (Fullerton, CA, USA) in the Laboratory of Capillary Chromatography and Electrophoresis at the Chemistry Institute of the University of São Paulo. The samples of the manipulated capsules and the standards of RSV were performed in triplicate. The areas under the peak were compared. The purity obtained was 87 ± 1.1% on average (coefficient of variation 1.2%).

### 2.2. Laboratory Tests

Laboratory tests were performed with biological samples collected after a 12 h fast. Venous blood samples were collected to obtain serum samples for biochemical analysis and whole blood for RNA extraction. Total cholesterol, triglycerides, HDL-cholesterol, and glucose were obtained by commercial colorimetric-enzymatic methods. LDL cholesterol was calculated using the Friedewald equation. The measurements were performed using the automated equipment Dimension RxL from Siemens Healthcare Diagnostics Inc. (Newark, DE, USA) with dedicated reagents. Insulin was analyzed by a chemi-luminescence assay using automated equipment Immulite 2000 from Siemens Healthcare. HOMA-IR was calculated using insulin and glucose levels. Sirt-1 serum concentration was determined with the ELISA kit from Uscn Life Science, Inc. (Wuhan, Hubei, China). Sirt-1 samples before and after interventions were analyzed in duplicate and in the same ELISA plate (coefficient of variation of 12% according to the manufacturer). esRAGE concentration was determined using the ELISA kit from the B-Bridge International (Santa Clara, CA, USA) using the Multiscan FC plate reader (Thermo Fischer Scientific, Vantaa, Finland). All tests were performed according to the manufacturers’ instructions.

### 2.3. Sirt-1 and RAGE Expression

Gene expression of Sirt-1 (Hs01009005_m1, Applied Biosystems; Foster City, CA, USA), flRAGE (00542592_G1), and esRAGE (HS00542584_G1, Applied Biosystems) [18] were evaluated pre-inclusion and postinclusion, according to the protocol. Total RNA was obtained using the TRIZOL reagent (Life Technologies, Waltham, MA, USA) from whole blood collected into an EDTA tube. cDNA synthesis was made with the Superscript II kit (Life Technologies) using 1ug from total RNA in a final volume of 20-µL reaction, according to the manufacturer’s instructions. The housekeeping gene was glyceraldehyde 3-phosphate dehydrogenase (GAPDH) (Hs02758991_g1). The reaction mix was prepared using 5 µL of the Universal Master Mix (Life Technologies), 0.5 µL of primers and probes mix (20×), and 2.5 µL of cDNA diluted samples (1:5). The PCR reaction was performed according to the following protocol: enzymatic activation for 2 min at 50 °C, initial denaturation for 10 min at 95 °C followed by 40 cycles of denaturation for 15 s at 95 °C, and annealing for 20 s at 60 °C. The reactions were run in triplicate and relative expression levels were calculated by normalizing the targets to the endogenously expressed housekeeping GAPDH gene. The results included the ratio between pre-intervention and postintervention values expressed in arbitrary units (AU).

### 2.4. Statistical Analysis

The sample size of 48 patients with 24 subjects per treatment arm was determined to yield a power of 80% with a 5% significance level to detect a 30% difference in Sirt1 plasma concentrations. Eligible female and male subjects were randomly assigned in a 1:1 ratio with the use of computer-generated random numbers to receive either RSV or CR. Pre-intervention and post-intervention variables were summarized with the use of descriptive statistics. All variables were analyzed descriptively. For the continuous variables, data are expressed as mean ± standard deviation (SD). Student *t* tests for comparisons between pre-interventions and post-interventions were performed for variables with normal distribution, which was verified by the analysis of the equality of variances (Folded F). Depending on the result of this analysis, the Pooled method (variances with *p* ≥ 0.05) or the Satterthwaite method (variances with *p* < 0.05) was used. The Spearman rank correlation method was used for correlations between variables. The level of significance was set at *p* < 0.05. The statistical software used was SAS version 9.3 (SAS Institute, Cary, NC, USA).

## 3. Results

**Figure 1:**
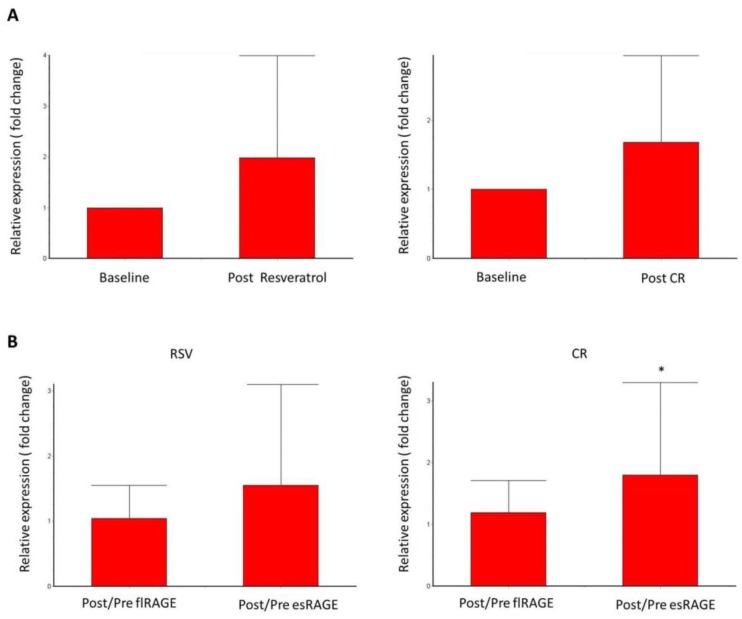
Real-time RT-PCR of Sirt-1 (**A**), esRAGE and flRAGE relation (**B**) after 30 days of caloric restriction or resveratrol intervention. Relative expressions (fold change) of mRNA transcripts were obtained by normalizing GAPDH gene. RSV: resveratrol, CR: caloric restriction. * *p* < 0.05.

Clinical features and laboratory data of participants before and after 30 days of intervention (CR and RSV groups) are shown in Table 1. For the RSV group, we observed increased serum concentrations of total cholesterol and non-HDL and HOMA-IR score. The other variables analyzed did not show any statistically significant differences after resveratrol administration. No side effects were reported. For the CR group, we observed that the average caloric intake for the 24 participants was 922.21 ± 27.37 kcal/day. Decreases occurred in weight, abdominal circumference, total cholesterol, HDL, non-HDL, and LDL. Serum concentration of Sirt1 was increased after both interventions, but showed no difference between study groups. The serum levels of esRAGE remained unaltered after interventions and no differences between groups were observed. Gene expression of Sirt1 was increased in both interventions without a difference between RSV and CR groups (*p* = 0.64). The relative expression of RAGE isoforms (post/pre) showed that esRAGE was increased after interventions, and flRAGE remained unchanged after interventions (Figure 1). esRAGE expression was about 57% higher than flRAGE in both groups but was statistically significant only in CR (*p* = 0.02). Positive correlations were observed between Sirt1, esRAGE, and flRAGE gene expressions in both groups. Sirt1 expression correlated with esRAGE expression (*r* = 0.86, *p* < 0.0001) and with flRAGE expression (*r* = 0.57, *p* < 0.0001) in the RSV group. In the CR group, Sirt-1 expression correlated with esRAGE expression (*r* = 0.71, *p* < 0.0001) and with flRAGE expression (*r* = 0.57; *p* = 0.0001). In the CR group, serum concentrations of esRAGE were correlated with esRAGE gene expression (*r* = 0.33, *p* = 0.04) and with Sirt1 gene expression (*r* = 0.32, *p* = 0.05). In the RSV group, serum concentrations of Sirt1 were negatively correlated with flRAGE expression (*r* = −0.30, *p* = 0.04).

## 4. Discussion

In this study, we compared the 30-day effects of RSV supplementation and CR in healthy slightly overweight individuals on Sirt-1 and RAGE isoform expression and serum levels. The important finding of the present study is that both RSV supplementation and CR stimulated Sirt-1 serum concentrations and CR elevated esRAGE mRNA production. 

The molecular mechanisms by which CR confers metabolic benefits are not entirely clear, but have been at least partly attributable to the regulation of energy homeostasis by Sirt-1 activation. Sirt-1 is an evolutionary conserved family of deacetylases and ADP-ribosyltransferases that directly regulates glucose and/or fat utilization in metabolically active tissues [19]. Howitz et al. [20] identified RSV as an activator of Sirt-1 and it has been suggested as a CR mimetic in the improvement of metabolic health [21]. Our results show that both interventions could directly induce increases in Sirt-1 expression at transcriptional and translational levels.

A transcriptional increase was also observed for esRAGE isoform after both interventions. RAGE is a multi-ligand receptor member of an immunoglobulin superfamily of cell-surface molecules. RAGE activation may be important for initializing and maintaining the pathological process that results in various diseases [22,23]. esRAGE has been the object of intense clinical research. The generation of soluble receptor isoforms represents an important mechanism to regulate aberrant receptor signaling in biological systems [24]. Soluble forms of RAGE seem to prevent ligands to interact with RAGE or other cell surface receptors [25]. esRAGE has an activity that neutralizes the AGE action and protects vascular cells against the activation of the cell-surface receptors and the AGE harmful positive loop of regulation [23,26]. Kierdorf et al. [27] have proposed that soluble RAGE does not act as a simple competitor but attenuates the activation of flRAGE by disturbing the preassembly of the receptor on the cell surface. Interactions between both RAGE molecules occur via the V and C1 domain, which enables the soluble RAGE to interact with membrane-bound flRAGE. The resulting hetero-multimers does not have competent signaling [27]. Decreased levels of esRAGE and/or increases in flRAGE are thought to enhance RAGE-mediated inflammation [18]. Prediabetic and diabetic patients exhibit lower esRAGE plasma levels and gene expression, which are inversely related to markers of inflammation and atherosclerotic risk [28]. Low levels of esRAGE have also been related to diastolic dysfunction [29]. Therefore, esRAGE could be a potential protective factor against the occurrence of cardiovascular disease. Our results show that esRAGE expression was approximately 57% higher than flRAGE expression after interventions. The relationship between CR or RSV and RAGE was previously demonstrated in experimental studies in which both significantly reduced RAGE mRNA transcripts [30,31]. However, little is known about CR and RSV interactions with esRAGE. In addition, the regulatory mechanism of the alternative splicing of esRAGE remains unknown. Alternative splicing is a regulated process that is mainly influenced by the activities of splicing regulators such as serine/arginine-rich proteins (SR proteins) or heterogeneous nuclear ribonucleoproteins (hnRNPs) [32]. Liu et al. [33] demonstrated the existence of hnRNP A1 in the splicing complex of RAGE and showed its involvement in the regulation of RAGE splicing. Splicing factor expression is known to be deregulated in senescent cells of multiple lineages and is a direct cause of multiple aspects of both aging and age-related disease in mammals [34]. Dietary restriction slows the accumulation of senescent cells [35]. Markus et al. [36] demonstrated that RSV could influence the splicing machinery. RSV had a selective effect on the levels of splicing factors inclusive of hnRNPA1. The increases in esRAGE expression may suggest a role for CR and RSV in the control of deleterious effects of the RAGE cascade. This increase of esRAGE stimulated by interventions may be supported by the positive correlation between esRAGE serum concentrations and Sirt-1 mRNA expression in CR and negative correlation of Sirt-1 serum levels and flRAGE gene expression in the RSV group. Serum levels of esRAGE remained unchanged, which may be due to the short follow-up time of 30 days. Despite the increase in gene expression, the steady-state protein levels in cells depend on the balance between their production and degradation. Protein ubiquitination is the central cellular process that directs protein degradation. Evankovich et al. identified that ubiquitin E3 ligase subunit F-box protein O10 (FBOX10), which mediates RAGE ubiquitination and degradation [37]. Possibly longer exposure to interventions could reverse the potential effect of ubiquitination on esRAGE proteins and increase serum levels of esRAGE.

Drugs like statins [38], methotrexate [39], metformin [40], and thiazolidinedione [41] were shown to increase soluble forms of RAGE. However, little is known about esRAGE alternative splicing induction by drugs and also about the increase of esRAGE in normal subjects. The elucidation of regulatory mechanisms of esRAGE is important from a clinical viewpoint and would provide a molecular basis for the development of drugs that can induce esRAGE and suppress cytotoxic effects of flRAGE.

The current study has some limitations, which include the small number of participants and the short follow-up period. However, in the literature, the studies citing esRAGE were obtained in patients with chronic degenerative disease. 

This study was the first one in healthy or slightly overweight subjects that showed an increase in esRAGE expression after CR and RSV interventions. Long-term randomized trials are needed to evaluate the possible clinical benefits of increased esRAGE expression in cardiovascular disease prevention.

In conclusion, this study shows that CR and RSV could effectively stimulate the increase in esRAGE expression in healthy subjects.

## Figures and Tables

**Table 1:** Clinical and laboratory characteristics of study participants before and after 30 days of resveratrol administration and caloric restriction.

	Resveratrol			Caloric Restriction		
	Baseline *n* = 24	30 days *n* = 24	*p*	Baseline *n* = 24	30 days *n* = 24	*p*
Age, years	58.46 ± 3.44			58.63 ± 3.65		
Weight, kg	83.01 ± 21.88	91.14 ± 17.77	0.328	69.13 ± 7.99	64.60 ± 7.30	0.002
Body mass index, kg/m^2^	27.61 ± 4.24	27.79 ± 4.38	0.370	25.84 ± 3.22	25.50 ± 3.21	0.083
Waist circumference, cm	96.82 ± 12.08	96.90 ± 11.36	0.457	94.27 ± 7.50	91.82 ± 7.12	0.011
Heart rate, bpm	64.61 ± 8.46	65.65 ± 8.22	0.269	62.50 ± 9.60	62.32 ± 10.51	0.902
Systolic BP, mmHg	131.46 ± 15.48	128.95 ± 15.44	0.660	129.73 ± 15.65	124.23 ± 12.81	0.109
Diastolic BP, mmHg	81.21 ± 10.81	81.95 ± 9.22	0.612	82.86 ± 10.96	79.36 ± 9.92	0.070
Total cholesterol, mmol/L	5.38 ± 0.85	5.64 ± 1.14	0.030	5.60 ± 1.12	5.25 ± 1.01	0.007
HDL-cholesterol, mmol/L	1.27 ± 0.35	1,25 ± 0.35	0.260	1.43 ± 0.47	1.35 ± 0.42	0.008
LDL-cholesterol, mmol/L	3.43 ± 0.68	3.61 ± 1.03	0.089	3.59 ± 0.93	3.37 ± 0.85	0.031
Non-HDL cholesterol mmol/L	4.11 ± 0.77	4.39 ± 1.07	0.010	4.17 ± 1.04	3.90 ± 0.98	0.025
Triglycerides, mmol/L	1.40 ± 0.73	1.68 ± 1.03	0.075	1.26 ± 0.70	1.15 ± 0.67	0.234
Glucose, mmol/L	5.26 ± 0.74	5.41 ± 0.79	0.165	5.20 ± 0.58	5.03 ± 0.32	0.118
Insulin, µUI/mL	7.85 ± 5.57	8.52 ± 5.67	0.066	6.71 ± 4.37	6.13 ± 3.16	0.428
HOMA-IR	1.66 ± 1.55	1.87 ± 1.70	0.038	1.49 ± 1.27	1.25 ± 0.74	0.275
Sirtuin1, ng/mL	1.06 ± 0.71	5.75 ± 2.98	<0.001	1.65 ± 1.81	5.80 ± 2.23	<0.001
esRAGE, pg/mL	255.78 ± 128.87	246.96 ± 115.32	0.800	246.67 ± 111.62	253.33 ± 116.81	0.857

BP: blood pressure, hsCRP: high-sensitivity C-reactive protein, HOMA: homeostatic model assessment, RAGE: endogenous soluble receptor for advanced glycation end products, AU: arbitrary unity.
